# The Crosstalk between the Blood–Brain Barrier Dysfunction and Neuroinflammation after General Anaesthesia

**DOI:** 10.3390/cimb44110386

**Published:** 2022-11-17

**Authors:** Xinxin Yang, Xiangdong Chen

**Affiliations:** Department of Anaesthesiology, Union Hospital, Tongji Medical College, Huazhong University of Science and Technology, Wuhan 430022, China

**Keywords:** general anaesthesia, blood–brain barrier dysfunction, neuroinflammation

## Abstract

As we know, with continuous medical progress, the treatment of many diseases can be conducted via surgery, which often relies on general anaesthesia for its satisfactory performance. With the widespread use of general anaesthetics, people are beginning to question the safety of general anaesthesia and there is a growing interest in central nervous system (CNS) complications associated with anaesthetics. Recently, abundant evidence has suggested that both blood–brain barrier (BBB) dysfunction and neuroinflammation play roles in the development of CNS complications after anaesthesia. Whether there is a crosstalk between BBB dysfunction and neuroinflammation after general anaesthesia, and whether this possible crosstalk could be a therapeutic target for CNS complications after general anaesthesia needs to be clarified by further studies.

## 1. Introduction

The continuous enhancement and refinement of surgery throughout the past decades could not have been achieved without the evolution of anaesthesia techniques and drugs. Advances in anaesthesia have made it possible to perform complex and long-term surgical procedures with safety and stability, making us believe that the development of anaesthesia can be considered one of the greatest achievements of medicine. Anaesthetics induce a controlled, reversible loss of consciousness by binding to specific receptors in the central nervous system (CNS), ensuring optimum conditions for patients undergoing surgery [1]. Clinical use of anaesthetics has generally been considered safe and effective before a growing number of studies in recent years have begun to question their safety. Several clinical and animal findings have proposed that anaesthetic drugs may induce long-term morphological and functional changes in the CNS with adverse effects [2,3,4,5]. Concerns about the potential neurotoxicity of anaesthetic drugs are growing [6]. Over recent years, an increasing number of studies have begun to focus on the close relationship between general-anaesthesia-induced neurocognitive dysfunction and neuroinflammation [7,8,9].

Normally, inflammation acts as a defensive response when exposed to destructive stimuli, but it becomes negatively impacted when it is abnormally amplified or becomes uncontrolled. As the target organ of general anaesthesia, the systemic inflammatory response could have a profound effect on the brain. Neuroinflammation as an inherent immune defence mechanism of the body plays an important role in maintaining the normal structure and function of the brain, but it is also an important factor contributing to neurodegenerative lesions and causing neuronal death [10]. It has been noted that the inflammatory response of the central nervous system is characteristic of almost all neurological disorders [11,12]. Under physiological conditions, a low expression level of inflammatory factors in the CNS is observed, and the expression levels of inflammatory cytokines such as IL-1β and tumour necrosis factor-α(TNF-α) are increased by varying degrees when there exists infection, surgical stimulation, or a stressful state in the CNS [13].

**Figure 1:**
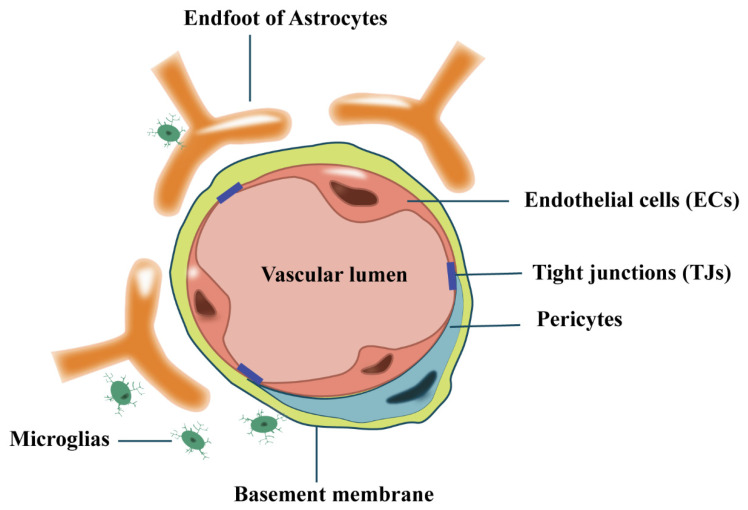
Schematic diagram of the blood–brain barrier (BBB). BBB consists of continuous endothelial cells (ECs) connected by tight junctions (TJs), which together with pericytes, astrocytes, microglia, and the surrounding basement membrane form a barrier.

To maintain homeostasis of brain tissue, a selective physical barrier is formed by a continuous layer of endothelial cells (ECs) connected by tight junctions (TJs), together with pericytes, astrocytes, microglia, and the surrounding basement membrane, which separates blood flow from brain parenchyma and regulates the movement of substances between the CNS and the periphery; this barrier is called the blood–brain barrier (BBB) (Figure 1). Research has indicated that dysfunction of the BBB is strongly associated with CNS diseases such as Alzheimer’s disease, Parkinson’s disease, amyotrophic lateral sclerosis, multiple sclerosis, and stroke [14].

The role of neuroinflammation in CNS complications associated with anaesthesia has received much attention and attracted the focus of researchers in recent years. It is hoped that the targeted therapy of neuroinflammation will improve brain dysfunction after anaesthesia, but further exploration of how anaesthesia triggers neuroinflammation is still underway. At this point, many studies have also observed that clinically used anaesthetics may disrupt the integrity of the BBB [15,16,17,18]. Thus, both neuroinflammation and BBB dysfunction have been observed following the use of narcotic drugs, yet the relationships between them, if any, have not yet been established.

In this review, we first briefly describe the function and structure of the BBB and then explore the effects of inflammation that affect the major components of the BBB. Next, we discuss microglia and astrocytes, which have been closely associated with the development of neuroinflammation, as well as the interactions between them. Particularly, we summarize recent advances in neuroinflammation and BBB destruction triggered by commonly used clinical anaesthetics, and propose potential future research directions as well as the possibility of improving anaesthetic-drug-related CNS complications through targeted control of neuroinflammation associated with BBB disorders.

## 2. The Blood–Brain-Barrier (BBB)

### 2.1. The Generation of BBB

The formation of CNS vasculature begins in early embryonic stages, and the interactions between neuroectodermal endodermal precursor cells as well as their correlated signal mechanisms play a key role in the development and maintenance of the CNS [19]. Endothelial tight junctions (TJs), nutrient transporters, numerous transcellular vesicles, and high expression of leukocyte adhesion molecules are present in blood vessels formed at the embryonic stage; however, TJs become more robust and complex, efflux transporters increase, and leukocyte adhesion factors are downregulated only when close contact is established with astrocytes and pericytes, achieving structural and functional maturation of the BBB [20,21], and astrocyte-derived sonic hedgehog (Shh) can impart different properties to BBB ECs from other tissue ECs [22]. Simultaneously, binding of pericytes to ECs triggers TGF-β production that maintains BBB permeability and produces extracellular matrix as well as extracellular matrix-expressing N-calcineurin [13,23], being a dynamic component of the BBB, the extracellular matrix regulates the structure and function of the BBB by affecting cell–cell and cell–matrix interactions [24], enhancing endothelial–pericyte interactions and further increasing pericyte binding on ECs, which are essential for maintaining BBB homeostasis [25]. All of the ECs, pericytes, astrocytes, and the basement membrane between them contribute to the BBB which is essential to maintain the function and integrity of the BBB [26].

### 2.2. Endothelial Cells (ECs)

**Figure 2:**
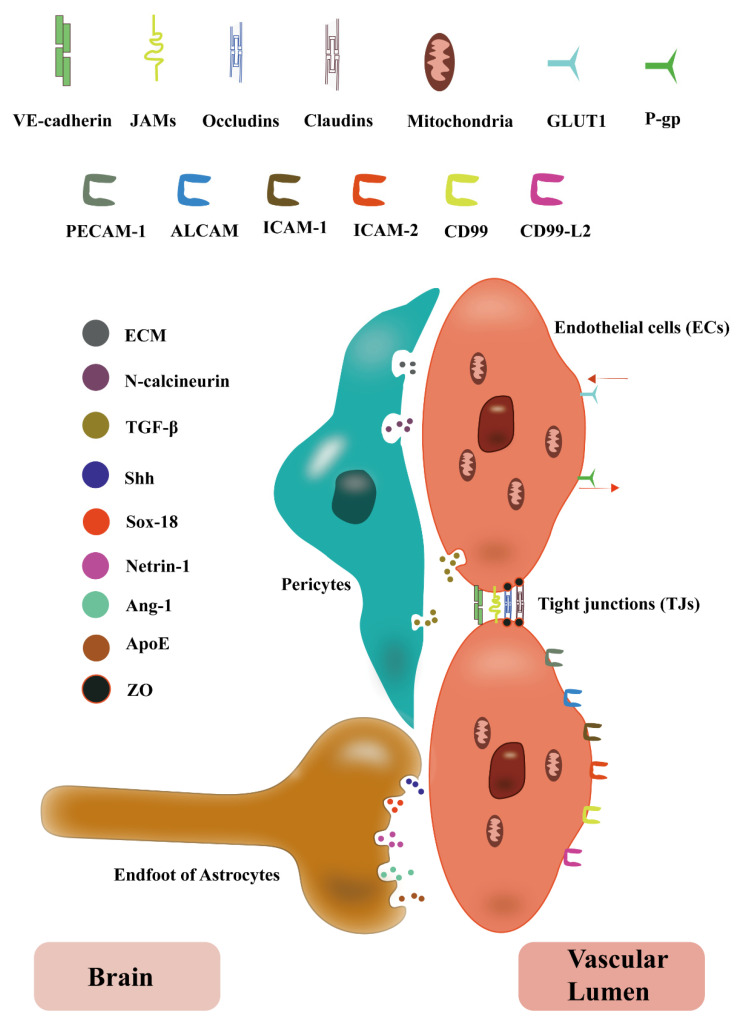
Structure and function of the main components of BBB. BBB ECs have a characteristic expression that differs from other ECs: a large number of mitochondria in the cytoplasm, characteristic inward transporters such as GLUT1 (glucose transporter protein 1), and outward transporters such as P-gp, as well as expression of adhesion molecules such as PECAM-1, ALCAM (activated leukocyte cell adhesion molecule), ICAM-1, ICAM-2, CD99, and CD99-L2, together with a specific VE-cadherin (vascular endothelial calcium adhesin) expression between ECs. Pericytes secrete TGF-β and N-calcineurin involved in maintaining BBB structure. TJs are mainly composed of claudins, occludins, and junctional adhesion molecules (JAMs), where claudins and occludins proteins are connected to the cytoskeleton by ZO proteins. Astrocytes are involved in maintaining BBB homeostasis through the secretion of Shh, Sox-18, Netrin-1, Ang-1, and ApoE.

The ECs are modified squamous epithelial cells of mesodermal origin participating in the formation of the vessel wall, anchored to the basement membrane with the help of cell adhesion molecules [27]. The structure and function of ECs on the BBB differ from those of other tissues (Figure 2), the existence of polarity in BBB ECs controls the directional movement of ions, molecules, and immune cells from the circulation to the CNS [28]. Moreover, BBB ECs have more mitochondria than those from peripheral tissues [29]. The presence of TJs between ECs constitutes a unique barrier characteristic of BBB which reduces paracellular transport, while transcellular transport is also hampered by the loss of fenestrations and reduced transcytosis [30,31]. Beyond this, BBB ECs have specific inward (e.g., glucose transporter protein 1) and outward transporters (e.g., P-gp (P-glycoprotein)) [14]. Concurrently, a marked downregulation of leukocyte adhesion molecule expression in BBB ECs restricted the entry of immune cells into the CNS [32], while several important adhesion molecules such as PECAM-1, activated leukocyte cell adhesion molecule, ICAM-1, ICAM-2, CD99, and CD99-L2 were expressed to participate in the migration of leukocytes on BBB ECs [33]. These specificities of BBB ECs facilitate the selective movement of substances between peripheral tissues and the CNS, and provide an effective barrier to the brain, with participation in maintaining a stable microenvironment for neurons.

In addition, vascular endothelial calcium adhesin resides between ECs (Figure 2); it regulates the shape, polarity, and lumen formation of ECs and is involved in the maintenance of vascular integrity and permeability through intracellular signal pathways and transcription factors as well as regulating ECs transcription and protecting ECs from apoptosis [34,35], which plays a key role in maintaining the integrity of BBB ECs [27].

### 2.3. Tight Junctions (TJs)

TJs between ECs constitute a unique barrier property of the BBB [36]. The presence of TJs causes an asymmetric distribution of ECs apical and basolateral cell membranes contributing to control of the permeability of the paracellular pathway across the BBB [37]. TJs consist of several different transmembrane proteins such as claudins, occludins, and junctional adhesion molecules (JAMs) (Figure 2) [38]. There are 25 known members of the claudin family with tissue-specific expression, claudin-1, -3, -5, -11, and -12 are mainly expressed in the CNS, furthermore, claudin-3, -5, and -12 are expressed in brain ECs where they participate in the maintenance of BBB function [39]. Occludins were the first TJ protein identified, and they form tight TJs through extracellular loop interactions, while the intracellular loop interacts with the band of ZO protein [26].JAMs belong to the CD2 subgroup of the Ig superfamily, and the main ones expressed on TJs in human BBB are JAM-A and JAM-C [40,41]. BBB JAMs can interact with integrin molecules expressed on the surface of various leukocytes, including T lymphocytes [42], which suggests that they may be involved in the migration of leukocytes in the BBB. TJ proteins are connected to the cytoskeleton via a multi-structured domain scaffold ZO protein [43]. ZO proteins contain three PSD-95/discharge/Zonula occludens-1 (PDZ) domains at their N termini, src-homology-3domain, and a region homologous to guanylate kinase [44]. ZO-1 interacts with the C-terminus of claudins through the PDZ-1 domain, and PDZ-2 and PDZ-3 mediate the interaction with occludin and JAMs [45,46].

### 2.4. Pericytes

The Pericytes are mesodermal-derived cells covering the CNS capillaries to regulate vascular stability, diameter, cerebral blood flow, and extracellular membrane protein secretion [13,47]. The BBB ECs and their associated pericytes both produce TGF-β, which is involved in regulating the maintenance of BBB properties and possess functional TGF-β receptors [48,49]. TGF-β signal in pericytes triggers the production of extracellular matrix molecules such as laminin, however, among BBB ECs, the TGF-β signal induces calbindin-2 (also known as N-calbindin) to promote pericyte adhesion (Figure 2) [50]. A defective TGF-β signal can lead to a detachment of pericytes from the CNS vasculature and lead to an increased BBB permeability and haemorrhage [51]. The above studies implied that the close association between BBB ECs and pericytes contributes to the regulation of trans-endothelial migration of leukocytes in homeostatic conditions as well as being involved in the maintenance of BBB homeostasis.

### 2.5. Astrocytes

One of the major cell types in the CNS, astrocytes are derived from radial glial cells [52], modulating the permeability of the BBB by forming firm contacts with the surface of CNS vessels through transmembrane anchoring proteins such as β-myotonic dystrophy protein and aquaporin 4 [53]. Astrocytes are known to play an important role in the acquisition and maintenance of BBB barrier properties and immune function via autocrine signals (Figure 2) [54]: a. The secreted glycoprotein Shh binds to the Patched-1 receptor at the surface of BBB ECs and engages in CNS-related morphogenesis through smooth molecule-induced signals [22,55]. b. Secretion of Sox-18 regulates the expression of claudin-5 in BBB [56]. c. The netrin-1 signal is secreted to regulate the expression of TJ molecules and inhibit the expression of CAMs [57]. d. The secreted Ang-1 binds to the receptor tyrosine kinase Tie-2 located on the surface of ECs, promotes angiogenesis, upregulates TJ molecules, and maintains BBB stability [58]. e. Secreting ApoE-containing lipoprotein particles takes part in maintaining the integrity of the BBB [59,60].

## 3. BBB and Inflammation

BBB strictly monitors the peripheral environment and regulates the entry of inflammatory factors, cells, and other substances into the CNS. The cellular and non-cellular components that exist in the BBB play their respective functions while working in concert with each other to maintain BBB homeostasis. Any of these components, directly or indirectly affected by inflammation, can lead to disruption of the BBB. It has been proven that activation of pro-inflammatory cytokines or enhanced pro-inflammatory responses can also directly impair the structure of BBB by increasing the permeability of ECs and disrupting the ZO-1 cell–cell border [61]. Recent studies have reported that inflammatory factors from the periphery entering the CNS after BBB fracture can further contribute to the development of neuroinflammation [62,63]. The disruption of BBB stability is closely associated with the development of neuroinflammation; next we delve into the role played by the various components of the BBB in the development of neuroinflammation and the changes they may undergo in the inflamed state.

### 3.1. Damage to ECs Is a Key Component of Neuroinflammation

**Figure 3:**
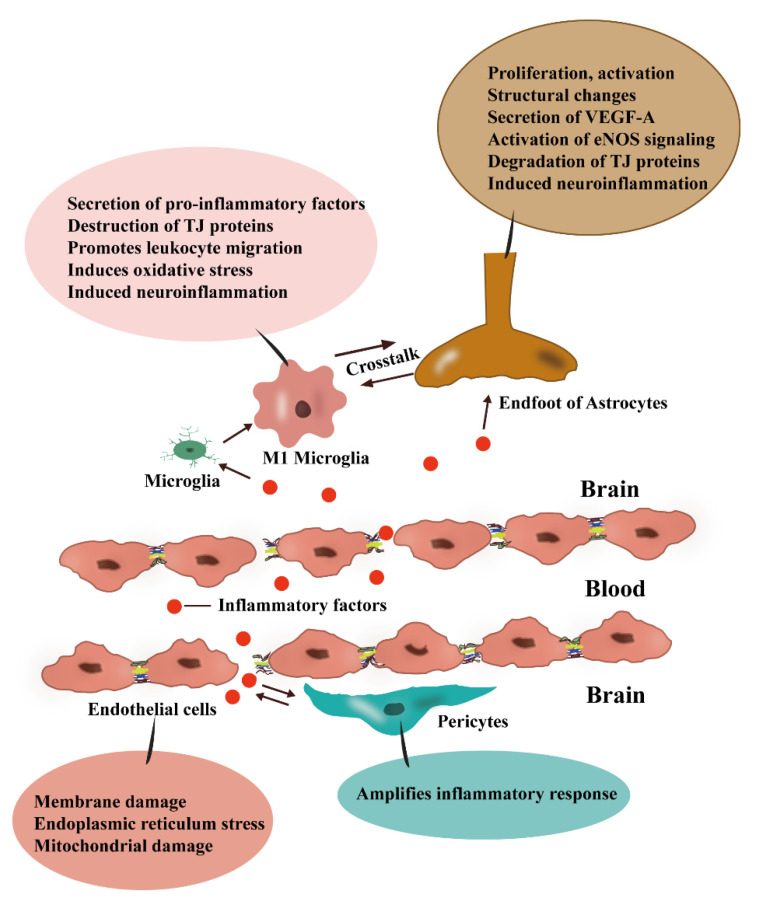
Schematic representation of BBB destruction due to inflammatory factors. Inflammatory factors can cause membrane damage, endoplasmic reticulum stress, and mitochondrial damage in ECs, disrupting TJs between ECs, leading to astrocyte proliferation activation, structural changes, secretion of VEGF-A, activation of eNOS signaling, degradation of TJ proteins, and ultimately neuroinflammation, at which point pericytes can amplify the inflammatory response. When inflammatory factors act on microglia, they can contribute to the activation of MI microglia which can lead to the secretion of pro-inflammatory factors, destruction of TJ proteins, promotion of leukocyte migration, induction of oxidative stress, and triggering of neuroinflammation. During this process, there is also a crosstalk between microglia and astrocytes.

Pro-inflammatory cytokines are accessible to the CNS directly through specific receptors and transporters on the surface of ECs crossing the BBB or the periventricular zone of the BBB [64]. Lipopolysaccharide (LPS) is an immunogenic component of Gram-negative bacteria and is widely used to model systemic inflammation. It was found that LPS could exert direct toxic effects on BBB ECs through repressing the activity of the outward transporter P-gp and inducing the secretion of matrix metalloproteinases, leading to cell membrane damage, endoplasmic reticulum stress and mitochondrial damage in BBB ECs, and ultimately triggering apoptosis (Figure 3) [65,66]. Meanwhile, inflammatory factor IL-1β may disrupt the integrity of the BBB by disrupting intercellular junctions and intercellular matrix adhesion of ECs [67]. In this section, we found that inflammation can have particularly severe effects on ECs via diverse mechanisms and that activation and dysfunction of BBB ECs in response to inflammatory stimuli are currently considered initial events in the development of neuroinflammation [68]. Therefore, disruption of ECs may be a key link in neuroinflammation associated with BBB dysfunction.

### 3.2. Inflammation Disrupts the Components of TJs

It has been revealed that inflammatory factors IL-1β, IL-6, IL-9, IL-17, IFN-γ, TNF-α, and CCL2 contribute to the destruction of TJs (Figure 3) [67,69,70,71]. As claudin-5 is the most important TJ protein associated with BBB selective permeability, it has been found that inflammation can lead to degeneration, downregulation of claudin-5 expression, and discontinuous distribution on the plasma membrane of ECs with further BBB disruption [67,72,73]. Apart from claudin-5, degradation of the occludin has been observed in LPS-induced systemic inflammation [74], and a more recent study has also shown that peripheral inflammatory cytokines reduce ZO-1 expression [75]. These studies tell us that the inflammatory state directly affects various aspects of the BBB TJs in a very critical way and further leads to BBB dysfunction through the disruption of the TJs.

### 3.3. Pericytes Expand the Inflammatory Response

One study observed that pericytes are a key source of neuroinflammation in cocaine-mediated neuroinflammation [76]. Pericyte-derived inflammatory mediators can also exert an enhanced inflammatory response and regulate the transport of immune cells to the CNS, playing a role in the maintenance of local inflammation [77]. Growing studies have also shown that the role of pericytes in promoting inflammatory responses can further lead to BBB destruction (Figure 3) [78,79]. Thereby, we know that when pericytes detect inflammatory stimuli, they can further amplify neuroinflammation and cause the destruction of the BBB.

In summary, inflammation may not only act directly on the various components of the BBB resulting in its destruction but may also trigger further neuroinflammation by acting on the BBB components. Of these, ECs are the primary targets for inflammation, which disrupts EC homeostasis and eventually triggers apoptosis, then, interrupts the connections between ECs by disrupting the extracellular matrix and degrading TJ proteins that lead to increased permeability of BBB, while pericytes mainly play a role in amplifying inflammation. Astrocytes, a key component of the BBB, are not discussed here, as we will explain later in the section “Glial Cells and Neuroinflammation”. Next, we turn our attention to astrocytes and microglia.

## 4. Glia Cells and Neuroinflammation

In the CNS, the occurrence of inflammation is mainly mediated by the activation of glial cells, especially astrocytes and microglia, which have been shown to cause prolonged activation leading to synaptic depression and cognitive dysfunction [63,80], neuroinflammation [63,81], and ultimately neurodegeneration [82,83].

### 4.1. Astrocytes as Mediators between Peripheral Inflammation and Neuroinflammation

Astrocytes are the dominant glial cells in the CNS numerically and play a key role in the maintenance of CNS homeostasis and related processes such as immune regulation through autocrine and paracrine signals [84]. Astrocytes have been shown to influence BBB permeability and infiltration of peripheral immune cells during the immune trigger or inflammatory phase [85]. Endotoxin-induced peripheral inflammation can also cause astrocytes proliferation, activation, altered end-foot structure, and other related gene expression alterations which collectively or indirectly lead to BBB destruction [86,87]. Inflammatory factors can increase BBB permeability by facilitating VEGF-A secretion from astrocytes, activating eNOS signal in ECs, and decreasing TJ protein occludin and claudin-5 expression, leading to inflammatory factors entering the CNS triggering neuroinflammation (Figure 3) [69,88]. Additionally, it has been found that the astrocytic protein S100 calcium-binding protein β (S100β), which is widespread in the brain, acts mainly as a neurotrophic or supportive factor when it is lowly expressed [89]; however, when expressed at elevated levels it may directly cause neuronal damage [90] and may also further activate microglia and astrocytes [91] and eventually induce reactive oxygen species (ROS) in microglia [92,93].

In addition to directly damaging the BBB, the above study suggests that inflammatory factors produced by peripheral tissues may also cause adverse effects on ECs and TJs through astrocytes, further promoting the development of neuroinflammation. Astrocytes may play a role in amplifying inflammation and act as a focal point for peripheral and neuroinflammation, and the astrocyte protein S100β is probably a biomarker for the development of neuroinflammation.

### 4.2. M1-Type Microglia Can Facilitate the Development of Neuroinflammation and Can also Disrupt the BBB

Microglia, as the major phagocytes in the brain, maintain brain homeostasis by engulfing cellular debris, absorbing harmful substances, and removing pathogens or necrotic cells. However, inflammation can prompt morphological changes in microglia and the upregulation of their specific expression of inflammatory signal receptors such as Toll-like receptor 4 (TLR-4) [94,95], which in turn activate microglia and further induce the development of CNS neuroinflammation, especially in hippocampal tissue [96,97]. Activated microglia have distinct functional phenotypes, including classically activated M1 microglia and alternatively-activated M2 microglia, which exert cytotoxic or neuroprotective effects, respectively [98].

M1 microglia can produce a variety of pro-inflammatory molecules, including but not limited to inflammatory cytokines, inducible nitric oxide synthase, nitric oxide, TNF-α, reactive oxygen species, and IL-6. Some studies have shown that inflammation-activated microglia can cause BBB destruction [99,100]. The massive release of inflammatory cytokines enhances the damage and destruction of the BBB through interactions with the BBB, including disruption of TJs activity, increase in paracellular permeability, promotion of leukocyte migration, and induction of adsorptive endocytosis, directly contributing to the inflammatory onset and damage of the BBB (Figure 3) [64,101]. IL-6 decreases the levels of claudin-5 and occludin in cerebral microvasculature [102]. Nitric oxide synthase decreases ZO-1 expression and increases BBB leakage through nitric oxide production [103] and the formation of peroxynitrite [104]. Alternatively, ROS irreversibly destroy cellular lipids, proteins, and DNA, which ultimately leads to cell death [105] and provides a common trigger mechanism for many downstream pathways that directly target and damage the BBB, such as oxidative damage, tight junction modifications, and matrix metalloproteinase activation [106], which in turn disrupt BBB homeostasis.

M2 microglia, on the other hand, phagocytose cellular debris and inhibit the development of inflammatory responses, facilitating the recovery and reduction of BBB injury [107,108]. M2 microglia have been shown to produce several anti-inflammatory cytokines such as IL-10, IL-4, and IL-13, thereby attenuating inflammatory damage to the BBB. IL-10 downregulates deleterious ROS-producing enzymes and/or upregulates antioxidant pathways to hinder the occurrence of ROS in ECs [109]. IL-4 and IL-13 directly promote phenotypic polarization of M2 microglia [110] and also inhibit the secretion of various pro-inflammatory mediators such as IL-6, IL-1β, TNF-α, and ROS [111,112].

Therefore, we know that activation of microglia with M1-type microglia predominance promotes the development of neuroinflammation and is closely related to the disruption of BBB homeostasis, while M2-type microglia predominance exerts a protective effect against the CNS.

### 4.3. Crosstalk between Microglia and Astrocytes

Interestingly, astrocytes and microglia do not independently act in the development of neuroinflammation, the crosstalk between astrocytes and microglia is very significant (Figure 3). Astrocytes indirectly activate microglia by inducing microglia CCR2 overexpression through the CCL2-CCR2 signal pathway, and blockade of CCR2 expression can attenuate inflammatory responses in microglia and improve cognitive function changes induced by neuroinflammation [113]. However, at the same time, astrocyte activation depends on microglia to a large extent. Activated microglia can also be induced to generate neurotoxic astrocytes via the complement cascade (C5, C3, and C1q) [114,115]. Recent studies have shown that microglia can also activate astrocytes [116,117]. Elimination of early microglia activation in hippocampal tissue diminishes long-term hippocampal astrocyte activation induced by etomidate [118]. It has also been demonstrated that microglia activated by endothelial cells and microglia activated by astrocytes have different phenotypes [119].

This information hints to us that there may be an interaction between microglia and astrocytes, and that there may be a “switch” involved in the balance between neuroinflammation and functional homeostasis in the brain.

## 5. Anaesthetics

General anaesthesia has been considered completely reversible in the past, and it was thought that although anaesthetic could cause significant changes in consciousness, it did not leave residual effects. However, there is growing evidence that general anaesthesia is not simply an “immediate reversible condition” but can affect neuronal function and disrupt CNS homeostasis, with acute and even long-term effects on the CNS [120,121,122]. To better treat anaesthesia-related CNS complications, we must master the mechanisms of their occurrence before targeting treatment. Multiple studies have demonstrated that anaesthetics modulate microglia activation in a time- and dose-dependent manner, triggering neuroinflammation and leading to undesirable CNS effects [123,124,125,126]. However, the specific mechanism of neuroinflammation due to general anaesthesia is not clear yet, and this issue awaits further studies addressing the neurotoxicity of anaesthetics. Subsequently, we will list some commonly used clinical anaesthetics for their role in the development of neuroinflammation and BBB dysfunction.

### 5.1. Propofol

Propofol is an ultrashort-acting intravenous anaesthetic drug [127] that causes increased Cl inward flow and hyperpolarization of neurons through binding to GABA-A receptors, ultimately leading to patient unresponsiveness to external stimuli [128,129]. Propofol was observed to cause apoptosis in CNS astrocytes in a cell-based assay, while a single dose of propofol was observed to inhibit microglia function and cause paradoxical behavioural manifestations in depressed mice, these studies revealed that propofol acts on glial cells interfering with brain homeostasis and neuroinflammation as well as being associated with decreased neurocognitive function [130,131]. It is also recently noted that propofol evokes severe neurotoxicity and is closely associated with the destruction of the BBB due to inflammation and injury of ECs [132]. The outcomes of the proteomic analysis suggest that propofol can negatively affect blood–brain barrier function by interfering with oxygen metabolism, DNA damage recognition, and response to stress [133]. ECs exposed to propofol also exhibit lower resistance and increased permeability, suggesting increased BBB permeability [15]. Additionally, it has been suggested that the disruption of BBB permeability in the developing brain by propofol also has long-term effects in adulthood [134].

These studies have allowed us to understand that propofol application has a long-term and profound effect on neuroinflammation and BBB disruption, but the exact mechanisms and connections are still unclear to us, and we expect that more studies will follow to focus on and investigate this issue to promote perioperative brain function homeostasis.

### 5.2. Inhalation Anaesthesia

Some early studies suggested that inhaled anaesthetic drugs could exert a cerebral protective effect by inhibiting BBB destruction [135,136,137]. However, studies in recent years have drawn different conclusions. ECs of rats exposed to the inhaled anaesthetic sevoflurane had significantly flattened luminal surfaces, showed ageing-related BBB damage, and weakened or disrupted BBB-associated tight junctions, thus disrupting brain homeostasis and perturbing neuronal function [18]. Hu et al. also noted that sevoflurane exposure exacerbated surgical stimulation-induced decrease in occludin expression and increase in matrix metalloproteinase protein expression, thereby exacerbating the damage to the BBB [138]. At the same time, clinically concentrated isoflurane leads to an immediate and significant increase in membrane fluidity in various membrane systems [139] and reversibly causes concentration- and time-dependent morphological damage to BBB ultrastructure and a significant decrease in tight junction protein occlusion protein expression ultimately leads to an increase in BBB permeability [140]. Research in the recent two years has also observed a correlation between sevoflurane inhalation anaesthesia and neuroinflammation. It was found that sevoflurane induced neuroinflammation by inhibiting PI3K/Akt/mTOR pathway signal [16,141] and the infusion of NAD-dependent deacetylase protein Sirtuin 3 into the hippocampus via a viral vector suppressed neuroinflammation and improved anaesthesia- and surgery-induced cognitive dysfunction [142]. These findings suggest that sevoflurane inhalation can cause cognitive impairment and is closely associated with hippocampal neuroinflammation.

To sum up, we noted a significant association between both inhaled anaesthetics and BBB destruction as well as neuroinflammation, however, there are no specific studies suggesting a link between the occurrence of BBB destruction and neuroinflammation mediated by inhaled anaesthetics. Is there a causal relationship or a reciprocal causal cascade amplification effect between these two? We believe this to be a question worth exploring and the underlying mechanisms require further exploration.

### 5.3. Opioids

Opioids mainly act on the central nervous system and are widely used in clinical anaesthesia for their analgesic effects. Opioid receptors are available in microglia, one study showed that morphine induced a dose-dependent decrease in the viability of BV-2 microglia and mouse primary microglia in an opioid-receptor-dependent manner, which triggered neuronal apoptosis [143]. It was also found in vitro that morphine application enhanced the LPS-induced release of inflammatory cytokines from microglia [144]. In addition to this, opioids can also affect microglia activity by binding to the innate immune receptor TLR4-related myeloid differentiation factor-2 (MD2) [145]. A significant increase in microglia Toll-like receptor 4 (TLR4) mRNA and protein expression was observed in morphine-exposed adolescent rats and was significantly associated with neuroinflammation. Interestingly, in rats, morphine-mediated microglia TLR4 activation was also gender-specific, with females showing a greater specificity for morphine [126,146]. In vitro, morphine triggers the activation of NOD-like receptor protein 3 (NLRP3), inflammatory vesicles, and inflammation in BV-2 microglia. Similarly, Peter et al. applied pharmacological and genetic approaches which observed that morphine induces NLRP3 inflammatory vesicles and subsequent IL-1β release in the spinal cord, that result in the subsequent development of long-term chronic pain. Furthermore, morphine also maintains the activation of NLRP3 inflammatory vesicles through the sustained release of damage-associated molecular patterns in a positive feedback manner [147,148,149]. Besides, it has been shown that opioids inhibit astrocyte synthesis and cause cellular hypertrophy as well as increase ROS concentrations [150,151]. Opioids such as morphine have also been shown to alter tight junction protein expression, leading to the disruption of BBB [152].

It is clear that opioid use in clinical anaesthesia is strongly associated with neuroinflammation. Moreover, this effect is achieved by activation of microglia, yet further studies are needed to confirm whether opioid-derived neuroinflammation is associated with BBB destruction. 

### 5.4. Different α2-Agonists

α2-agonists are a commonly used sedative drug in clinical anaesthesia, acting on widely expressed α2-adrenergic receptors in the CNS to exert sedation, analgesia, bradycardia, hypotension, and hypothermic effects [153]. Unlike other anaesthetics, α2-agonists exert an anti-inflammatory and neuroprotective effect in the CNS. In cultured microglia activated by LPS, the commonly used α2-agonist dexmedetomidine (DEX) inhibited the production and release of inflammatory mediators and cytokines including iNOS or NO, IL-1β, and TNF-α in a dose-dependent manner [154,155], while impeding microglia activation and enhancing microglia phagocytosis [156,157]. Apart from this, various assays have shown the anti-inflammatory and neuroprotective effects of DEX mediated by miRNAs. The enhanced miRNA-381 and inhibition of the Egr1/p53 pathway induced by DEX in mice undergoing sevoflurane anaesthesia were associated with apoptosis of hippocampal neurons, DNA [158] injury, neuroinflammation, and lower cognitive impairment [159], with antagonistic effects in different pathological models of neuroinflammation, ischemia-reperfusion injury, and anaesthesia-induced neurotoxicity [154,160,161,162]. MiR-155 is a critical miRNA in BBB-associated neuroinflammation and has a negative regulatory effect on BBB [163]. Paeschke et al. observed miRNA-155 upregulation in the hippocampus, cortex, and plasma expression in a time-dependent manner during LPS-induced neuroinflammation, while DEX treatment significantly attenuated this effect [164].

By reviewing the studies related to α2-agonists, it is clear that it exerts neuroprotective effects through different mechanisms, and we note that there may be a correlation between the inhibition of neuroinflammation and the function of the BBB for this effect. However, there are no relevant studies to confirm this association. Future studies are needed to explore such possibilities and contribute to perioperative brain homeostasis.

## 6. Conclusions and Future Directions

In the “Anaesthesia” section, we summarized some commonly used perioperative anaesthetic drugs such as propofol, inhaled anaesthetics, opioids, and α2-agonists for their roles in neuroinflammation and BBB function. We noticed a tight association between several anaesthetics and the development of neuroinflammation and BBB dysfunction, except for α2 agonists, which exerted a positive effect. In clinical practice, multiple types of anaesthetics are often used in a certain order, however, we discussed only a few of the most commonly used anaesthetics and limit ourselves to the effects of a single drug here. Besides those, we mainly referred to some animal and cellular studies, where the combination of multiple drugs may lead to different outcomes. Extra studies are needed to investigate the effects of the combination of multiple anaesthetics on neuroinflammation and BBB function in clinical situations.

Altogether, a single administration of the commonly used anaesthetics of propofol, inhalation anaesthetics, and opioids can induce neuroinflammation as well as BBB dysfunction among animal and cellular studies. As discussed earlier, we outlined the crosstalk between neuroinflammation and BBB dysfunction. However, there are still no relevant studies to prove whether there is a crossover between BBB dysfunction and neuroinflammation caused by anaesthetic drugs. The crossover of neuroinflammation and BBB dysfunction provides new insights into the central role of anaesthetics as well as opens up new and exciting breakthroughs and possibilities for studying CNS complications associated with general anaesthesia, which is the innovation of this paper.

## Data Availability

Not applicable.
